# Protein surface representation and analysis by dimension reduction

**DOI:** 10.1186/1477-5956-10-S1-S1

**Published:** 2012-06-21

**Authors:** Heng Yang, Rehman Qureshi, Ahmet Sacan

**Affiliations:** 1 Center for Integrated Bioinformatics, School of Biomedical Engineering, Science and Health System, Drexel University, 3120 Market Street, Philadelphia, PA 19104, USA

## Abstract

**Background:**

Protein structures are better conserved than protein sequences, and consequently more functional information is available in structures than in sequences. However, proteins generally interact with other proteins and molecules via their surface regions and a backbone-only analysis of protein structures may miss many of the functional and evolutionary features. Surface information can help better elucidate proteins' functions and their interactions with other proteins. Computational analysis and comparison of protein surfaces is an important challenge to overcome to enable efficient and accurate functional characterization of proteins.

**Methods:**

In this study we present a new method for representation and comparison of protein surface features. Our method is based on mapping the 3-D protein surfaces onto 2-D maps using various dimension reduction methods. We have proposed area and neighbor based metrics in order to evaluate the accuracy of this surface representation. In order to capture functionally relevant information, we encode geometric and biochemical features of the protein, such as hydrophobicity, electrostatic potential, and curvature, into separate color channels in the 2-D map. The resulting images can then be compared using efficient 2-D image registration methods to identify surface regions and features shared by proteins.

**Results:**

We demonstrate the utility of our method and characterize its performance using both synthetic and real data. Among the dimension reduction methods investigated, SNE, LandmarkIsomap, Isomap, and Sammon's mapping provide the best performance in preserving the area and neighborhood properties of the original 3-D surface. The enriched 2-D representation is shown to be useful in characterizing the functional site of chymotrypsin and able to detect structural similarities in heat shock proteins. A texture mapping using the 2-D representation is also proposed as an interesting application to structure visualization.

## Background

The advent of new technologies has resulted in a massive expansion of the protein sequence and structure databases. This enables the characterization of the similarities of sequences and structures and identification of the location of functional sites. High throughput sequencing data analysis has opened up new applications and facilitated the study of proteins. The alignment of protein sequences and structures has been able to investigate convergent and divergent protein relationships; this has been facilitated by the exponentially increasing size of the available data. Traditionally, sequence analysis has been largely based on pairwise and multiple sequence alignments, which are algorithmically based on dynamic programming [1,2]. Heuristic approaches have been proposed to speed up the alignment against large sequence databases [3,4].

Unlike protein sequences, protein structure analysis has not yet enjoyed a widely accepted comparison or search method. The Protein Databank (PDB) is a repository of 3-D protein and nucleic acids structures [5]. As of February 2012, there are nearly 80,000 protein structures available in the PDB. The increasing availability of this data brings computational challenges as well as opportunities. In order to make effective use of this data, there is a growing need for more sensitive and automated comparison, search, and analysis tools for protein structures.

Unlike sequence alignment, structure alignment captures information not detectable in a protein's sequence due to the nature of protein folding: two amino acids that are far away from each other in a protein sequence may be brought close together when the protein folds. Although three dimensional protein structures are determined by primary sequences, even large sequence variations (due to mutation) usually only cause minor and unimportant structural variations. Thus, evolutionary relationships are best detected at the structural level.

The computation of protein structure alignment is a computationally hard problem, due to the number of possible combinations of residue associations that can be used to generate corresponding translation and rotation matrices, and it is usually solved by heuristic approaches [5-9]. Despite active research and the availability of a growing number of methods, there is no widely accepted 3-D residue-based structural alignment method. Furthermore, most of the existing structural alignment methods focus only on the backbone chain to decrease the computational burden. However, this simplification causes the loss of important information contained on the surface.

**Figure 1:**
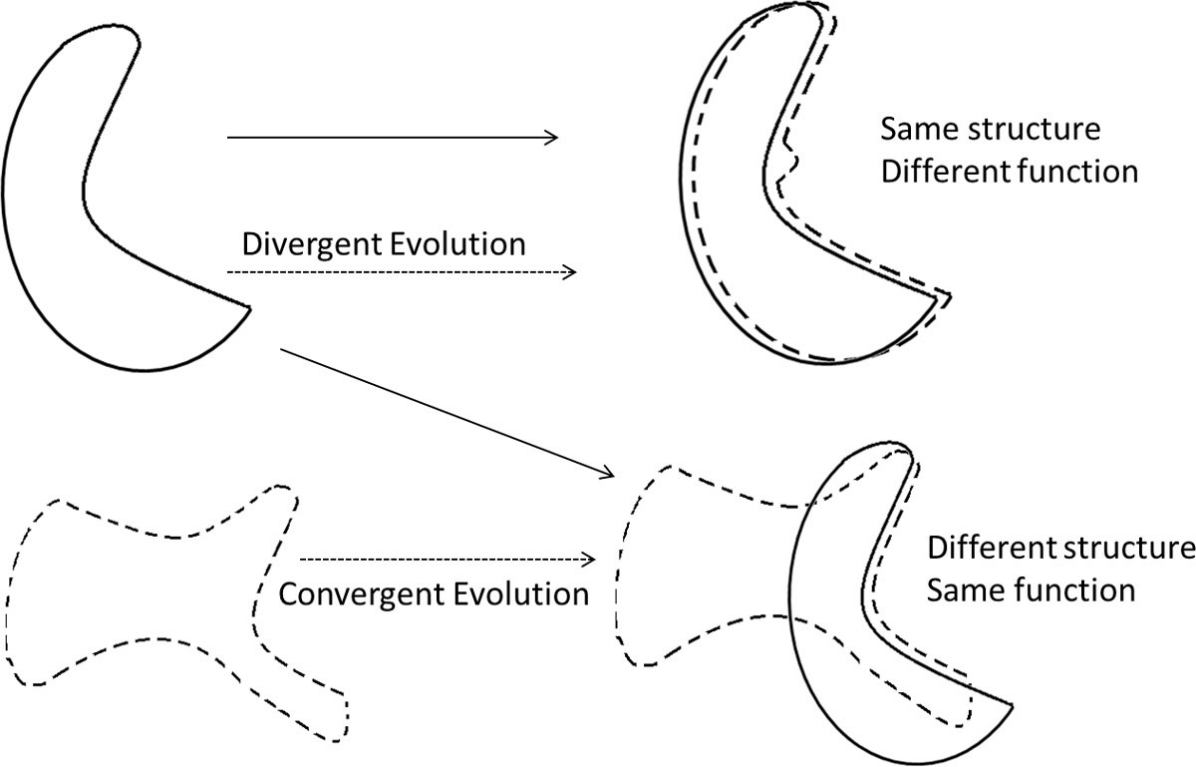
**Schematic illustration of importance of local surface characterization compared to structure comparison**: In the upper right-hand picture, two proteins that have almost the same overall structure are shown. Despite the highly similar structures, a small local difference in their binding site due to divergent evolution may cause these proteins to have different functions. In the lower right-hand picture, two structurally different proteins are shown in bold line and dotted line, respectively. Although these proteins differ in their global structure, by convergent evolution, they may share similar local binding sites and may have similar functions. (Adapted from [6]).

Protein structure uncovers more distant evolutionary relationships than protein sequence. However, we cannot neglect the fact that two different proteins' surface characteristics may converge through evolution and result in similar functions [6]. Two proteins might have different backbones and different overall 3-D structures, and still possess highly similar surface regions, giving them the ability to catalyze chemically equivalent reactions on similar substrates [7] (see Figure 1 for an illustration). Proteins that meet these conditions are likely experiencing convergent or divergent evolution. In the case of divergent evolution, two protein sequences or structures can mutate over time, but the surface characteristics must be conserved in order to maintain the specific function. In the case of convergent evolution, proteins with similar functions but different structures can evolve similar surface characteristics, causing non-homologous proteins to share similar active or binding sites [8,9]. The conservation of similar local sites on protein surfaces may not be detected by sequence or structure comparison, but the surface determinants can determine the common functionality, making surface based methods invaluable for protein functional annotation.

In addition to advancing the general body of functional knowledge for proteins, protein surfaces can also play a role in rational drug design. The analysis of protein surfaces could identify protein binding pockets so that the requirements for a given pharmaceutical compound's size and binding orientation can be determined [10]. Furthermore, knowledge of the protein conformation can help researchers develop specific pharmaceuticals for a given disease. This analysis can also assist in the investigation of protein-protein interactions and give researchers insight into the biological processes of the cell. For example, signal transduction is carried out by a cascade of protein-protein interactions, involving multi-peptide complexes that associate by surface complementarity. Moreover, the ligand binding sites act as a signal trigger that is usually located in the protein surface pockets. Once, the ligand binds to the protein's active site, it alters the protein's 3-D structure and thus triggers a certain response.

In this paper, we introduce a new method for the analysis and comparison of protein surfaces. We utilize a two dimensional representation that enables efficient computational storage and comparison. The reduced representation is optimized using dimension reduction methods, such that the geometric relationship of the atoms in 3-D is preserved in the 2-D representation. We demonstrate that this reduced representation captures biologically important information by characterizing an enriched 2-D map of a chymotrypsin protein and by comparing enriched image representations of heat shock proteins.

## Methods

First, we consider the problem of map generation as an error minimization problem, and utilize dimension reduction methods to perform this mapping. While the accuracy of earlier studies is limited by how close the shape of protein is to a sphere or ellipse, our approach attempts to address more complex shapes, as are present in almost all proteins. Secondly, we enrich the surface map with bio-chemical and geometrical properties, such as electrostatic potential, hydrophobicity and curvature, in order to facilitate functional analysis. While other surface features can also be mapped in a similar fashion as the method described here, we leave an exhaustive feature mapping to a future endeavor. Image registration is then applied on these feature-enriched images. Thirdly, we utilize the 3D-2D mapping to perform texture-mapping of arbitrary images back on the 3-D surface, to complement the existing graphical visualization options.

**Figure 2:**
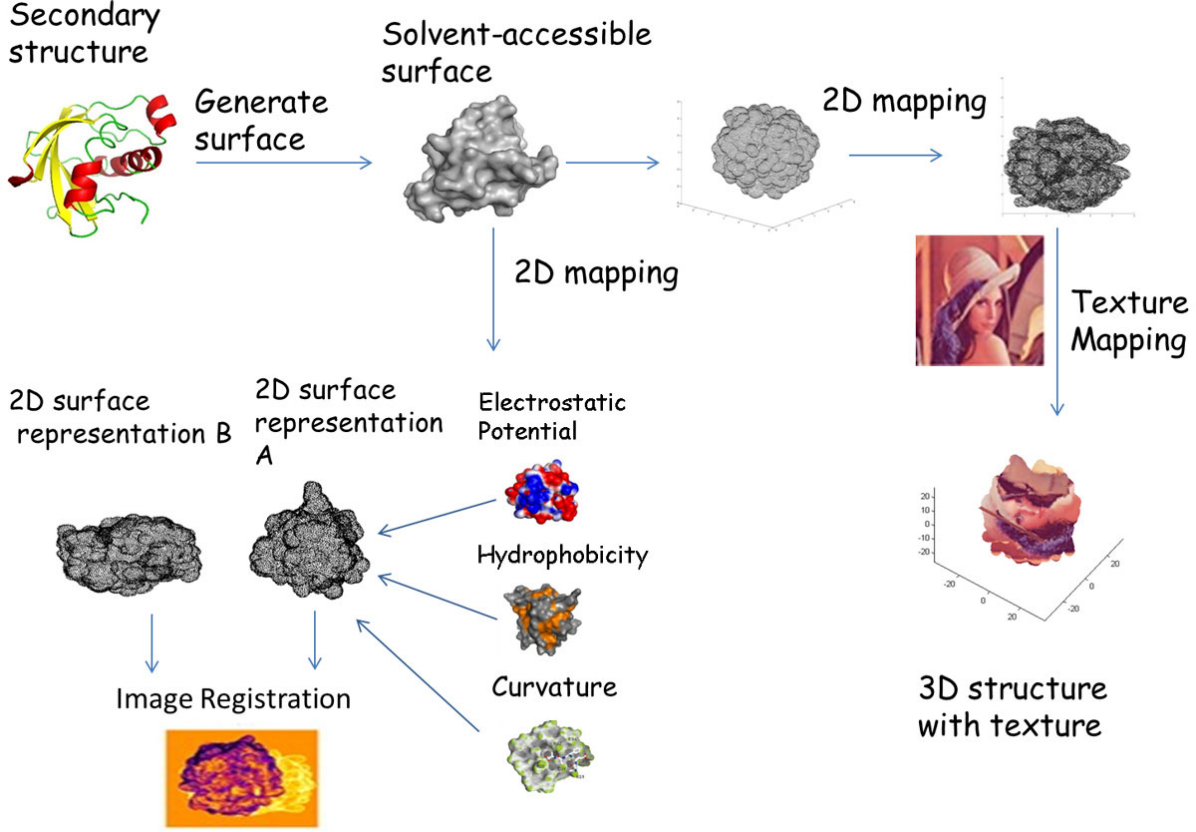
**Flow chart of the main tasks involved in this study**: The main workflow of this study starts with generation of the solvent excluded surface using the 3-D atomic coordinates and the generation of a 2-D mapping using dimension reduction. The reduced representation is enriched with geometric and biochemical features calculated from the 3-D surface points, such as electrostatic potential, hydrophobicity, and curvature. The enriched 2-D surface representation of proteins can then be compared using image registration methods. The 2D-3D mapping of the protein surface can also be utilized to texture-map arbitrary images back onto the 3-D surface, to aid in visualization.

Figure 2 shows a flow chart describing the workflow of the project. The atomic coordinates are extracted from the PDB file, and the solvent excluded surface is calculated [11]. We generated the 3-D surface using the MSMS program developed by Sanner [12], using a probe radius of 1.4 angstroms. The mapping of the 3-D surface points to a 2-D map is done using dimension reduction methods. After generation of this 2-D map, the workflow shows two directions; the first leading to feature-enrichment and comparison of proteins, and the seconds leading to texture-mapping for visualization purposes. In the former, the electrostatic potential, hydrophobicity and surface curvature are calculated and enriched onto the 2-D surface. Image registration is then used to maximally align the two surfaces to find the similarities. In the latter direction, texture mapping is applied to superimpose an arbitrary template image onto the 2-D surface. Each point on the 2-D surface is assigned the pixel value found where the point and pixel overlapped. The color value of each point is assigned back onto the corresponding point in 3-D. Delaunay triangulation is applied to the surface points in order to obtain a triangulated mesh for the evaluation of 3-D to 2-D mapping methods. Delaunay triangulation is commonly used to generate surface meshes from point clouds, the fundamental criterion of it is that the circum-circle of each Delaunay triangle contains no other point in its interior [13].

### Generation of the 3-D surface

**Figure 3:**
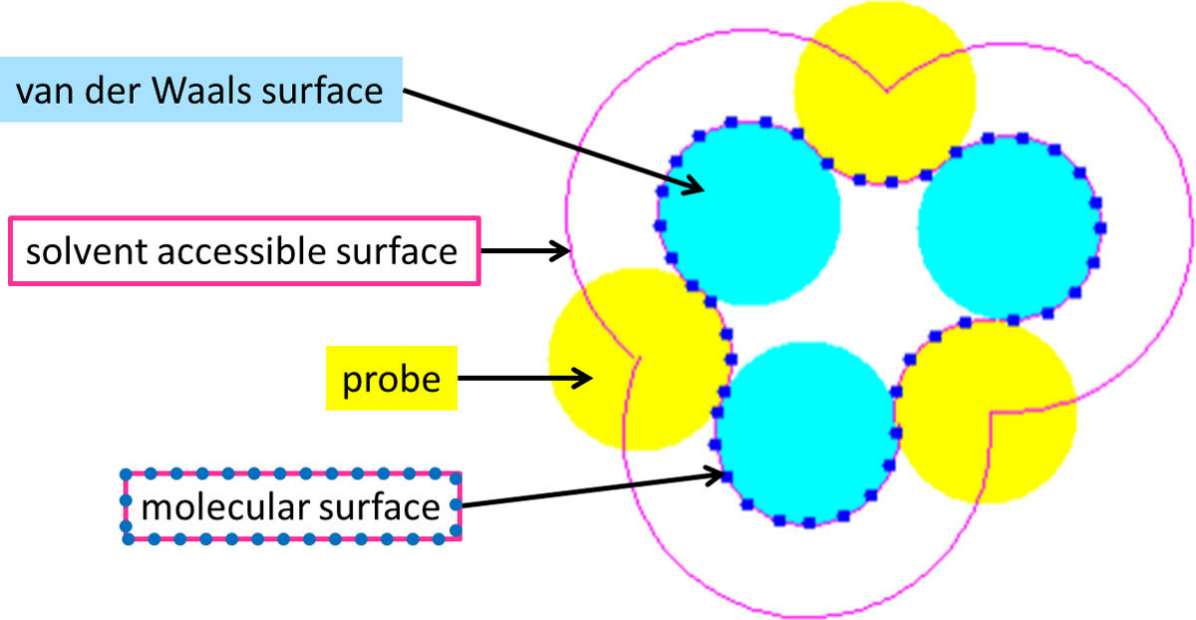
**Illustration of different protein surface definitions**: A van der Waals surface (cyan) is obtained by taking the union of the spherical atom surfaces defined by the van der Waals radius of each atom. Solvent accessible surface (pink) is defined by the path traced by the center of a probe (yellow) that is rolled around the protein. Molecular surface (solvent-excluded surface) is the set of points traced by the inward-facing part of the probe.

A number of different representations have been developed to describe the protein surface (See Figure 3). A classic representation is the solvent accessible surface, introduced by Lee and Richards [14]. The accessible surface can be determined by simulating a probe "rolling" on the surface. The path traced out by the center of the probe forms the solvent accessible surface.

Connolly developed a numerical algorithm to calculate the 3-D protein contour based on solvent-accessible surface method [11]. Later a surface triangulation method was developed by Connolly [15] which is based on subdividing the curved faces of an analytical molecular surface representation.

Connolly later introduced another representation of a protein's surface, called the Molecular surface (also known as the solvent-excluded surface or Connolly surface) [16]. Unlike the solvent accessible surface, which is considered the expanded van der Waals surface of the protein, the Molecular surface is defined as the inward-facing part of the probe that is rolling on the protein surface. In the present study, we utilize this surface representation of the proteins.

**Figure 4:**
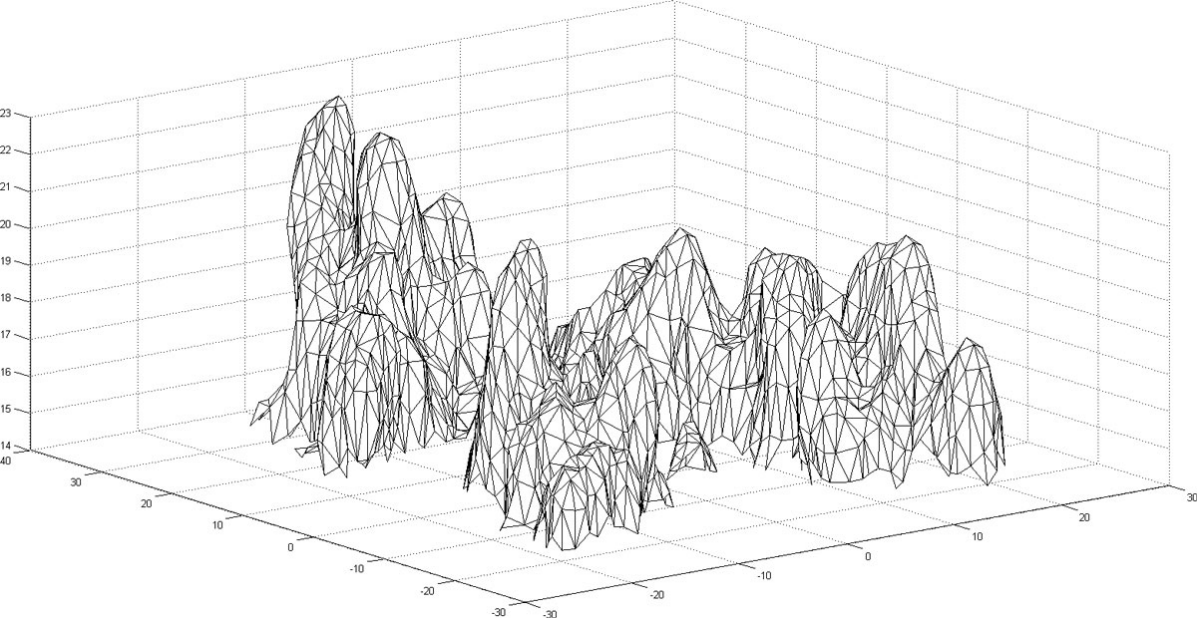
**Triangulated surface of a heat shock protein**. The excluded solvent surface points and their triangulation for a heat shock protein (PDB ID: 1kaz) are generated using the MSMS software [12]. Only a portion of the entire protein is shown for clarity.

Starting from Connolly's work, numerous methods have been proposed for surface representation, Sanner [17] introduced the idea of r-reduced surface and developed an efficient algorithm to compute the outer components of the surface. The molecular surface of a heat shock protein using Sanner's implementation is shown in Figure 4 as a triangulated surface. Staib [18] developed a mathematical surface representation by expansions of spherical harmonic functions, which can be used in analyzing surface curvatures, surface interaction, and surface visualization. While we have utilized the solvent excluded representation in this study, the approach introduced here can be extended to these other surface representations.

Protein surfaces generated by these methods have found use in a variety of visualization and analysis applications. Almost all popular macromolecular visualization programs now contain routines for the generation and visualization of different types of surface representations [19,20].

The difficulty of dealing with surfaces is apparent, in comparison to the more widely utilized primary sequence or backbone conformation, which possess numerous alignment methods. Due to the complexity of the surfaces and the lack of established methods for general-purpose analysis, most studies have focused on certain surface features, such as active or functional sites and structural motifs [21]. These sites are identified only around a local spatial proximity or surface patch and involve only a few highly conserved amino acids [22].

Approaches that have attempted to represent and analyze the entire surface have been geared toward extracting generic shape parameters that are not amenable to detailed characterization of surfaces. 3-D Spherical harmonics and Zernike descriptors have been used as feature vectors for protein structure comparison and similarity-based retrieval [23]. Geometric hashing has also been used for translation and rotation invariant comparison of sets of atoms [24]. Poirette [25] has used the genetic algorithm to compare two protein surfaces by searching for a translation and rotation matrix that brings the two surfaces together, maximizing the surface overlap.

Our approach to study the protein surfaces is based on finding an accurate representation of the protein surface in 2-D. Representing 3-D surfaces in 2-D is an old problem, most known in geography, where some terrain or the entire globe is shown on a map. This process has been coined "molecular cartography" for macromolecular structures [26,27]. These early studies in molecular cartography have remained isolated and have not gained a sufficient following. With the current study, we hope to bring the power and appeal of molecular cartography back to the attention of the protein structural analysis community.

The studies by both Fanning et al. [26] and Pawlowski and Godzik [27] have borrowed ideas from cartography studies and have applied similar projections used therein. Fanning et al. [26] have generated a contour map of the surface, in order to preserve some of the topographic features of the irregular protein shapes. They have used Mercator-like projection and Mollweide projection in order to investigate whether topographic features can provide antigenic determinants. Pawlowski and Godzik [27] have used an equal area sinusoidal cartographic projection (also known as the Mercator equal-area projection) as a simple surface representation to measure the map similarity of proteins.

Our approach is based on the method of representing the surface of a three dimensional object on several planar grids. A dimension reduction method (DRM) is a geometric technique that maps higher dimensional data into a lower dimensional space while preserving some geometrical properties of the higher dimensional data, such as the variance or inter-point distances. There are many different dimension reduction methods, but they generally fall into three categories: linear methods, global nonlinear methods, and local nonlinear methods.

Principal Components Analysis (PCA) is one of the classic linear dimension reduction methods. It attempts to find a linear mapping between high dimensional and low dimensional data using the principal eigenvectors of the covariance matrix of data [28] PCA is able to reveal the internal structure of the data in a way that best captures the data variance. However, since the principal eigenvectors rely mainly on the data dimensionality, PCA is not appropriate for reducing relatively high dimensional data or data that do not lie on a linear subspace [29].

Multidimensional Scaling (MDS) is a global nonlinear dimension reduction method. MDS constructs a dissimilarity matrix in high dimensional data points using Euclidean distance and tries to maintain the minimum distance errors. The stress function of choice is often Kruskal's stress or Sammon's stress measures [30]. Other global nonlinear methods that were investigated in this study include Stochastic Neighbor Embedding (SNE) [31], Isomap [32], and Stochastic Proximity Embedding (SPE) [33]. Like MDS, SNE attempts to preserve pairwise distances between data points in low dimensions, but the distance measure and the cost function are different from MDS, MDS measures the Euclidean distance between two points, while SNE measures their probability generated by the Gaussian kernel function, and the Kullback-Leilbler divergences are utilized to measure the probability difference. SNE is found to be more advantageous in preserving the local properties of a manifold. T-distributed Stochastic Neighbor Embedding (tSNE) is a variation of SNE; it uses student-t distribution instead of Gaussian as its cost function, and is able to avoid the "crowding problem" that often appears in SNE [34]. Isomap uses the geodesic distances among the data points in the original space to address the "Swiss roll" problem [29].

Locally-Linear Embedding (LLE) involves finding the nearest neighbors of each point and then determining weights for each point in order to express the point as a linear combination of its neighbors[35]. The weights are a set describing how much each neighbor contributes to determining the location of the given point. LLE then uses the set of weights to place the point in a lower dimensional space. Thus, in lower dimensional space, any given point is still described by the same weight function. LLE can run more quickly than Isomap when it uses sparse matrix algorithms, but cannot handle non-uniform sample densities as well [29].

### Evaluation of dimension reduction

While visual inspection of the 2-D mappings can provide insights into the maps generated by different methods, a more quantitative evaluation is needed for comparison. For a given dataset of protein structures, we evaluate each dimension reduction method based on its accuracy and speed in mapping the surface. The accuracy of a dimension reduction method is evaluated by its ability to preserve the spatial features and relationships among the points. We have defined two assessment criteria for area and neighbor preservation. The area score (ranging from -1 to 1) is calculated as the Pearson's correlation coefficient between the areas of the surface triangles in 3-D and in 2-D, and it measures the level of distortions induced by the mapping procedure. The higher the correlation value of a method, the better the method preserves the relative spatial distributions of the points. The neighbor score (ranging from 0 to 1) evaluates the ability of a method to preserve the neighborhood relationships among the points, and is calculated using the Tanimoto similarity coefficient of the connectivity matrices of all points in 2-D and 3-D. In 3-D, the connectivity matrix is obtained using the geodesic distances of the k-nearest neighbors of each point. In 2-D, the connectivity matrix is obtained using the Euclidean distance of the k-nearest neighbors of each point (we used k = 3). These connectivity matrices are then represented as linear bit vectors (with only 0 or 1 values) X and Y. The Tanimoto coefficient [36] of two bit vectors X and Y is defined as follows:

$$T\;\left(X,\;Y\right)=\frac{X\;\cdot Y}{||X|{|}^{2}+||Y|{|}^{2}-X\;\cdot Y}$$

The higher the Tanimoto coefficient, the better the method is at preserving the neighbors of the points.

### Feature enrichment with geometric and biochemical properties

Dimension reduction provides a mapping on to the 2-D space, and implicitly captures only the geometric properties of the original surface points. While the area and neighborhood properties of the points are preserved as much as possible under the constraints of the 2-D space, other geometric properties are mostly ignored. Note however, that we can enrich the mapped points with color channels representing additional information. Specifically, we associate a color channel for curvature information, thus capture additional 3-D geometric properties.

Even though geometric features of the protein surface are important functional determinants, the biochemical properties of the surface points are as important in determining binding interactions and enzymatic activity. We have investigated two biochemical features: electrostatic potential and hydrophobicity. The electrostatic potential plays an important role in indicating molecular interactions and protein folding. We have used the Poisson-Boltzman formulation to calculate the electrostatic potential at each surface point [37]. The hydrophobicity property has been one of the most used properties in studying protein structure and folding. For globular proteins, the hydrophobic side chains are usually buried inside the protein structure while the hydrophilic side chains are exposed to the water. We have utilized the Kyte-Doolittle hydrophobicity scale [38] to calculate the level of hydrophobicity of each surface point.

### Image registration

There are many registration methods available, and they can be classified in different ways such as feature-based and intensity-based methods. Feature-based methods use the common features to find the correspondence between two images. These features can be based on points, curves, or surfaces, each with a corresponding distance metric to facilitate the identification of feature associations between two images. The feature-based methods work well if the images contain salient patterns, such as corners or contours. Intensity-based methods rely on pixel intensities and have several different metrics, such as Normalized Cross Correlation of the pixel intensities, Mutual Information, and the Sum of Squared Differences [39].

## Results

In order to evaluate our approach and characterize the performance of different methods and parameter choices, we used both synthetic and real data. The synthetic data consisted of an idealized sphere with 162 equi-distant points triangulated into 320 triangles. The real data consisted of 8198 surface points generated from protein 1kaz which were triangulated into 16160 triangles.

**Figure 5:**
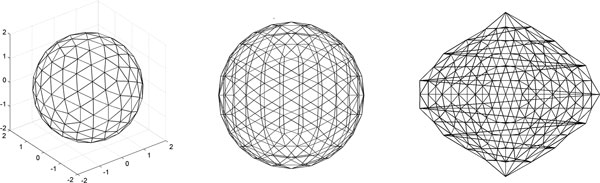
**Complete enclosed sphere and its 2D mappings**: An enclosed spherical surface (left) is mapped using PCA (middle) and using sinusoidal cartography (right). The PCA method causes a collapse of the sphere in 2-D and cannot preserve the neighborhood relationship of the surface points. Sinusoidal cartography avoids this problem to some extent (except for the points at the boundaries of the 2-D map), but is not able to preserve the area information of the 3-D surface polygons.

**Figure 6:**
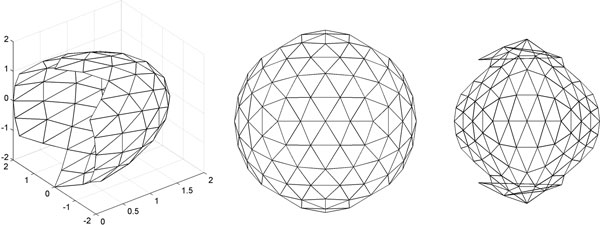
**Hemisphere and its 2D mappings**: A hemi-spherical surface (left) is obtained by cutting the sphere with a plane through its center. The hemisphere is mapped using PCA (middle) and using sinusoidal cartography (right). Both PCA and sinusoidal cartography are able to preserve the local geometric properties of the points better than the spherical mapping case.

Mapping of an idealized spherical surface using PCA and sinusoidal cartography [27] is illustrated in Figure 5. Note that due the inherent property of an enclosed 3-D surface, it is not possible to equally maintain geometric relationship of all the surface points. Notably, the points in 2-D would have different local neighbors than they had in 3-D. In order to alleviate this problem, we section an enclosed surface and consider each sub-surface separately (See Figure 6). Both PCA and sinusoidal cartography methods are able to better preserve the local geometric properties of the surface points being mapped.

In order to evaluate the performance of various dimension reduction methods, we mapped a triangulated hemisphere with equally distributed points. Table 1 shows the accuracy and time performance of each method. Isomap, SNE, LandmarkIsoMap, and Sammon had better area and neighbor scores than the other methods. Isomap had the best are score and second best neighbor score. Sammon had a slightly better neighbor score than Isomap. Among the top scoring methods, LandmarkIsomap had the best running time, followed by Sammon. Other faster methods did not have satisfactory area and neighbor scores. Table 1 also indicates that area score positively correlates with neighbor score, indicating that the failure of maintaining the same area is due to the triangle distortion in which the neighbor points have been moved away from each other. The exception to this was tSNE, which despite having a poor area score, had neighbor score comparable to the top scoring methods.

**Table 1:** Performance of dimension reduction methods for mapping a triangulated hemisphere

Methods	Area	Neighbor	Runtime (sec)
PCA	0.28	0.46	**0.002**
LLE	0.28	0.47	0.03
Laplacian	0.11	0.29	0.02
LLC	-0.01	0.02	1.16
AutoEncoderEA	0.01	0.11	2.83
SNE	**0.72**	**0.53**	6.81
SymSNE	0.03	0.01	6.75
CFA	0.27	0.12	4.36
GPLVM	0.28	0.46	0.3
NPE	0.28	0.47	0.03
LPP	-0.007	0.17	**0.004**
LLTSA	0.28	0.46	0.029
NCA	0.28	0.09	3.1
MCML	0.28	0.46	0.79
LDA	0.28	0.37	**0.005**
FactorAnalysis	0.24	0.21	**0.004**
tSNE	0.16	**0.53**	0.61
Isomap	**0.81**	**0.53**	0.29
LandmarkIsomap	**0.69**	**0.53**	0.06
ProbPCA	0.28	0.48	0.11
KernelPCA	0.04	0.23	0.007
MDS	0.28	0.46	**0.004**
DiffusionMaps	0.3	0.48	**0.006**
Sammon	**0.73**	**0.54**	0.1
Sinusoidalcartography	0.10	0.45	**0.003**

For each performance criteria, the top comparable scores are shown in bold. knn = 3 was used for the neighbor scoring.

Protein structures have naturally evolved to have more complex shapes, with pockets and protrusions. In order to evaluate the performance of the dimension reduction methods on real proteins, we performed the same experiment describe above for an idealized hemisphere, to a heat shock protein ATPase domain (PDB ID: 1kaz[40]), The protein is cut into six sections based on three orthogonal planes of the coordinate system. The section with the positive Y coordinates was chosen for this experiment, and contained 8198 points and 16160 triangles. The performance of the dimension reduction methods on this section is summarized in Table 2.

**Table 2:** Performance of dimension reduction methods for mapping a section of the heat shock protein ATPase domain

Methods	Area	Neighbor	Runtime (sec)
PCA	**0.69**	0.17	**0.002**
LLE	**0.66**	0.13	27.1
Laplacian	0.48	0.22	7.57
LLC	0.19	0.07	13.4
AutoEncoderEA	0.52	0.05	73.2
SNE	**0.69**	0.24	8824
SymSNE	-0.0012	0.002	7733
CFA	**0.69**	0.08	421.3
GPLVM	**0.69**	0.17	4477
NPE	**0.70**	0.13	23.4
LPP	**0.69**	0.13	20.56
LLTSA	**0.69**	0.17	13.8
NCA	**0.69**	0.08	178.8
MCML	**0.69**	0.16	1337
LDA	**0.69**	0.14	0.46
FactorAnalysis	**0.69**	0.16	0.02
tSNE	0.12	**0.36**	2295
Isomap	**0.70**	0.23	8396
LandmarkIsomap	**0.70**	0.23	1739
ProbPCA	**0.68**	0.01	10.18
KernelPCA	0.03	0.08	679.8
MDS	**0.69**	0.17	**0.002**
DiffusionMaps	**0.69**	0.18	4046
Sammon	**0.69**	0.18	3294
Sinusoidalcartography	0.10	0.2	**0.005**

For each performance criteria, the top comparable scores are shown in bold. knn = 3 was used for the neighbor scoring.

As expected from the more complex structure of the protein, the dimension reduction methods result in lower area and neighbor scores compared to the simpler hemisphere experiment above. 18 of the methods have an area score between 0.66 and 0.70. Among these, SNE, Isomap, and LandmarkIsomap have a neighbor score better than 0.23. The protein surface contained 50 times more points than the hemisphere, which significantly increased the running time for most of the methods.

**Figure 7:**
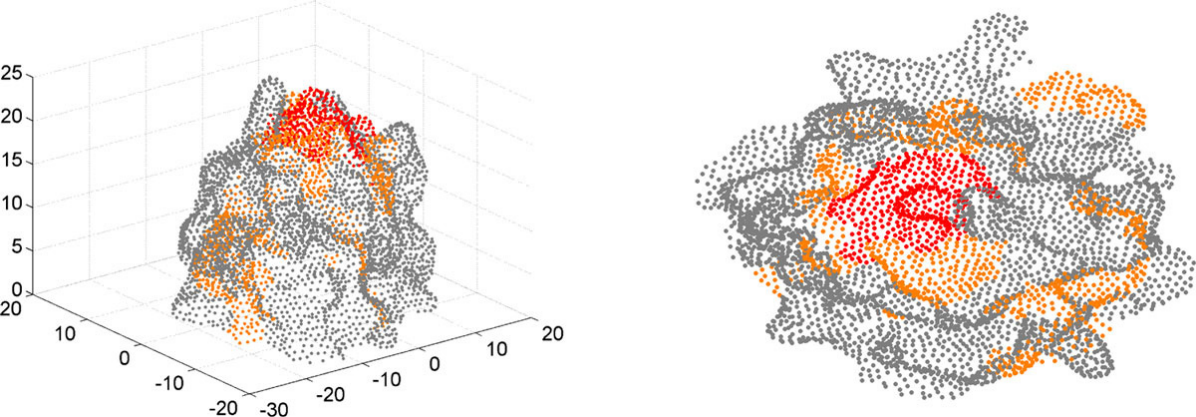
**Mapping the surface hydrophobicity of chymotrypsin**: A half-section of the chymotrypsin protein (PDB ID: 2ea3) in 3-D is shown on the left, as a surface point cloud, color-coded by hydrophobic (orange) and hydrophilic (gray) regions, and active site (red). A 2-D mapping of chymotrypsin half-section using Sammon mapping is shown on the right.

A visual inspection of the 2-D surface maps confirmed that the methods SNE, Isomap, LandmarkIsomap, and Sammon produced comparable maps that were superior to those generated by other methods (results not shown). We have used the 2-D maps produced by Sammon mapping for the downstream analysis described below. In order to evaluate the ability of our 2-D representation to capture the biologically important information, we conducted an experiment on a bacterial chymotrypsin (PDB ID: 2ea3) [41] Chymotrypsin is a serine protease in and has a well-characterized active site containing a catalytic triad. The section of the chymotrypsin with positive Z coordinates is used here, since that is section containing the active site. Figure 7 shows the surface of the chymotrypsin as a point cloud, color coded by the hydrophobic and hydrophilic regions in a gradient from orange to gray, respectively. The active site is highlighted in red. The 2-D surface mapping resulting from the Sammon method is shown on the right, with the mapped points colored in the same color value that they had in 3-D. It can be observed that the regions with distinct hydrophobicity are preserved in the 2-D map. Additionally, the points on the active site maintain their spatial relationship in the mapping. The active site can be seen to reside in a hydrophobic region, surrounded by hydrophilic atoms.

**Figure 8:**
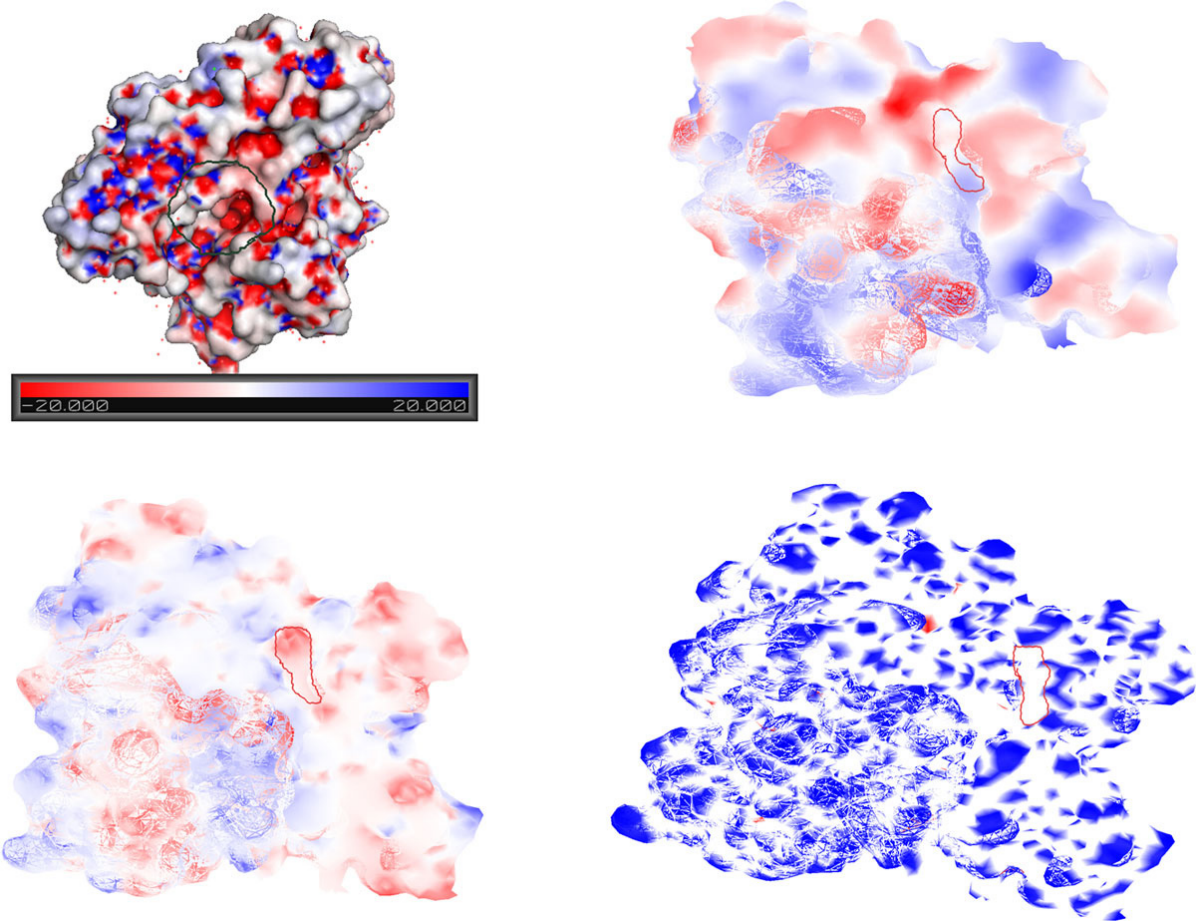
**Structure and mapped surfaces of **1kaz: The 3-D structure of a heat shock protein (PDB: 1kaz) is shown in the top-left, color-coded by electrostatic potential; negative, positive, and neutral regions are shown in blue, red, and white, respectively (image generated using Pymol [45]). Top right, bottom left, and bottom right figures show the 2-D map of the heat shock protein, enriched with hydrophobicity, electrostatic potential, and curvature information, respectively. Hydrophobic and hydrophilic regions are calculated using the Kyte-Doolittle scale are shown in red and blue, respectively. Convex and concave regions are shown in blue and white, respectively. The active site is denoted with a closed curve in each image.

In order to evaluate whether the 2-D representation of protein surfaces can be used for surface comparison, we conducted an experiment with two heat shock proteins (PDB IDs: 1bup[42] and 1kaz[40]). These proteins share a high sequence similarity (99% identity) and were chosen to simplify the visual inspection of their surfaces. Each protein is bisected by the x, y or z coordinate planes, resulting in six unique sections for each protein. In the examples below, only the positive Y coordinate sections are shown. Sammon Mapping is performed on the sections obtained from the two proteins. The proteins were then enriched with electrostatic potential, hydrophobicity and curvature information. The active site residues are obtained from the Catalytic Site Atlas [43,44] and mapped and highlighted onto the enriched surfaces, shown in Figure 8.

The hydrophobicity map reveals that the active site lies on a blue hydrophobic belt-shaped area. The electrostatic potential image shows the active site to lie in a red region of negative charge, and the curvature image shows it to be a concave valley enclosed by a bulged "mountain" structure. These observations conform to the relevant theory and also prove the accuracy of the feature enrichment as a descriptive guide for characterization of the active site.

Once the hydrophobicity, electrostatic potential and curvature models are generated, two (or more) protein surfaces can be compared. Note that the proteins are likely to be in different spatial orientations in the model database, thus an image registration needs to be performed to reorient and superpose them before similarities can be highlighted. We have left an investigation of automated image registration methods for a future study and have resorted to a manual image registration at present to ensure accuracy.

**Figure 9:**
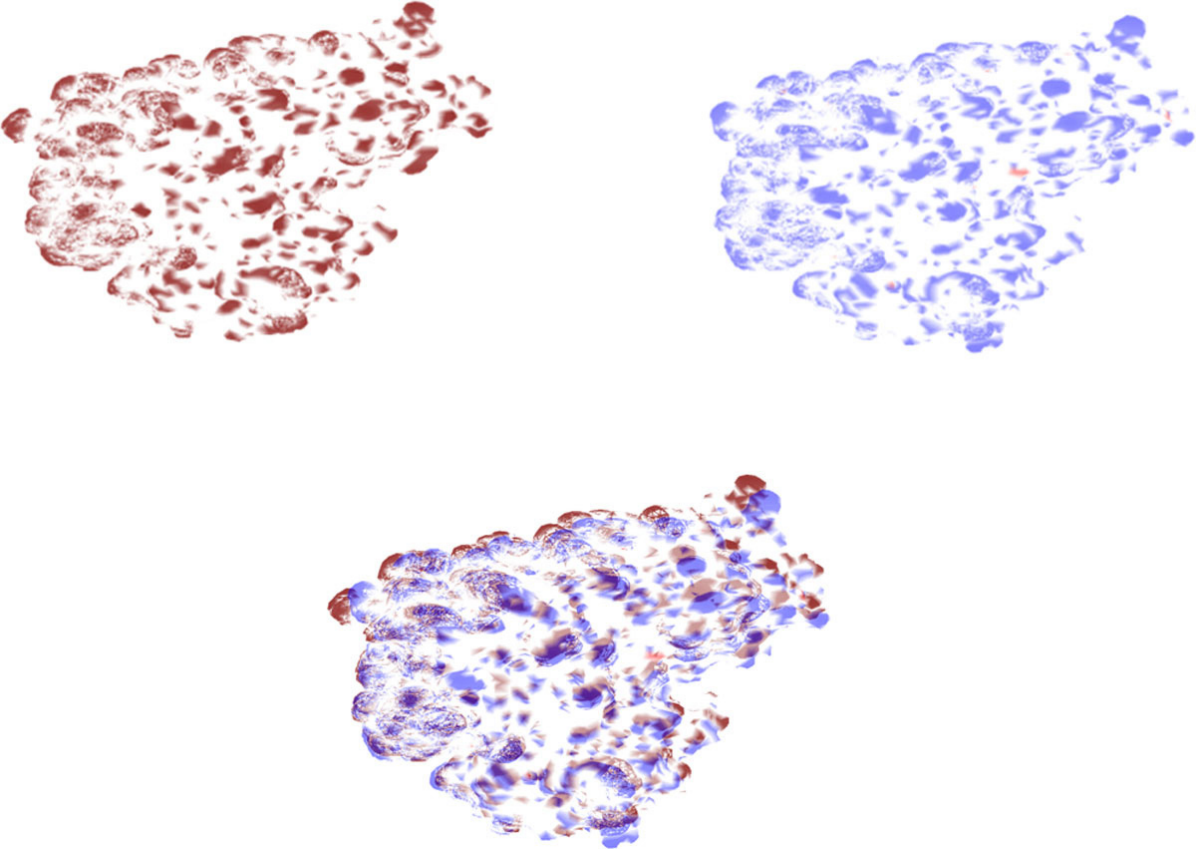
**Image registration of protein curvature maps from heat shock proteins **1kaz and 1bup: 2-D curvature maps of heat shock proteins 1bup and 1kaz are shown in the upper left and right, respectively. Superposition of their curvature maps is shown in the bottom figure.

The claim that our representation can capture structural similarity can best be demonstrated using the 2-D curvature maps of proteins. Figure 9 shows the 2-D curvature maps of heat shock proteins 1bup and 1kaz, and their superposition. The superposition shows a high degree of overlap between the two proteins, demonstrating that the 2-D representation contains sufficient information to identify structural similarities between proteins. Note that in a functional site identification context, the biochemical features would also be important determinants of similarity. Due to the difficulty of visual display and interpretation of multi-channel images, we have omitted combined feature maps from this presentation.

### Texture mapping

**Figure 10:**
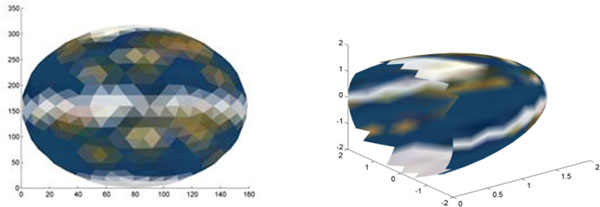
**Texture mapping on the hemisphere**: On the left, a 2-D map of a hemisphere obtained using PCA is overlaid with an image of the world map; the parts of the image not assigned to any points are not shown. 3-D texture mapping of the hemisphere using the assigned pixel values is shown on the right.

The 2-D mapping presented in here lends itself to an interesting visualization application. Specifically, the 2-D map of a surface can be overlaid with an arbitrary image to associate each map point with a pixel value from the image. Figure 10 shows the texture-mapping of the hemisphere with an image of the world. The blur in the image is due to the limited number of points we have used to represent a hemisphere. When the surface is redrawn with the assigned pixel values in 3-D, each triangle is colored using interpolation of the color values of its three points, to obtain a smooth image.

**Figure 11:**
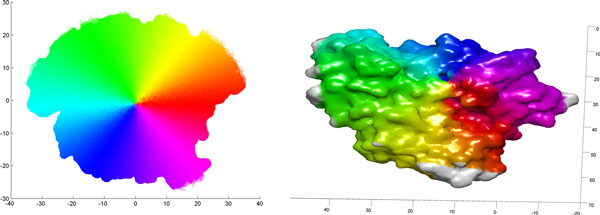
**Texture mapping on the protein**: On the left, a 2-D map of a half-section from a heat shock protein (PDB ID: 1kaz) is shown overlaid with an image of a color wheel. On the right, a 3-D texture map of the color wheel onto the protein surface is shown.

The process of texture mapping is repeated for a heat shock protein (PDB ID: 1kaz), where an image of a color wheel is texture-mapped on the protein (See Figure 11). The spatial neighborhood of the 3-D points is extremely well preserved, as seen from the continuous gradation of the colors in 3-D. We envision this texture mapping to be useful in transferring annotations that can more easily performed on a 2D mapping, back onto the 3-D surface.

## Conclusions

We proposed a 3-D to 2-D surface mapping method for protein surfaces using dimension reduction methods. Protein function is largely dependent on surface features, especially the functional sites. Surface features are reducible to protein structure, and ultimately to sequence information, but convergent evolution has produced proteins with dissimilar sequences and/or structures which nevertheless have similar surface properties and functions. Surface comparison is expected to identify protein function with greater efficiency than existing methods (sequence/structure comparison) by obviating complex structural analysis in favor of surface features, which have greater functional relevance.

Thus, surface comparison attempts to identify protein functional sites which are better predictors of protein function than sequence or structural features. We achieve surface comparison by mapping 3-D protein surfaces into 2-D through dimension reduction methods and enriching the 2-D representation with biochemical and geometrical features. Various dimension reduction methods are evaluated for their ability to accurately represent the protein surface and their computational efficiency. The alignment of pairs of protein models obtained by these methods is obtained through a manual image registration process. An automated registration process which quantifies the similarity of proteins and localizes the active site will be presented separately. Furthermore, the 3-D to 2-D mapping of surface points enables novel visualizations of protein structure and properties, including texture mapping in which the 2-D protein map is overlaid onto an arbitrary image. Future work, including the automatic implementation of the entire workflow described above, provides a clear path toward a protein function prediction system in which a query protein is ranked against a database of existing protein surface maps. Prediction of protein-protein interaction partners and interaction sites is possible through a similar system in which a query protein would be searched against a pre-built database of interaction sites, and their correlation used in making predictions.

## Competing interests

The authors declare that they have no competing interests.

## Authors' contributions

AS conceived of the study and coordinated the project. HY was the primary developer, who implemented the method and performed the experiments. RQ contributed to the design and test of the method. All authors participated in the analysis of the results. HY and RQ contributed to the writing of the manuscript. All authors read and approved of the final draft.
